# Synthesis and Biological Evaluation of the Anti-Melanogenesis Effect of Coumaric and Caffeic Acid-Conjugated Peptides in Human Melanocytes

**DOI:** 10.3389/fphar.2020.00922

**Published:** 2020-06-17

**Authors:** Kyeong-Yong Park, Jiyeon Kim

**Affiliations:** ^1^Department of Integrated Material's Development, CHA Meditech Co., Ltd, Daejeon, South Korea; ^2^Department of Medical Laboratory Science, College of Health Science, Dankook University, Cheonan, South Korea

**Keywords:** SK-MEL-2, melanin, α-MSH, tyrosinase, coumaric acid

## Abstract

Excessive pigmentation and reduced elasticity are the major skin problems that dermatologists and cosmetologists address. Compounds that inhibit melanin production might contribute to improving skin problems. In this study, we investigated whether coumaric acid- and caffeic acid-conjugated peptides might affect alpha-melanocyte stimulating hormone-induced melanin production, tyrosinase activity, and melanin synthesis-related gene expression in SK-MEL-2 human melanoma cells. Coumaric acid and caffeic acid showed no significant cytotoxicity, and they inhibited melanin production. In addition, coumaric acid- and caffeic acid-conjugated peptides suppressed tyrosinase activity more than arbutin, a known tyrosinase inhibitor. Quantitative real-time PCR (qRT-PCR) results also showed that both peptides inhibited the expression of melanin synthesis-related genes, *TYR*, *TYRP1*, *TYRP2*, and *MITF*. In particular, among the nine conjugated peptides tested, caffeic acid linked to a Gly-Gly-Gly linker and conjugated to the tripeptide, ARP, showed the greatest inhibition of gene expression in the qRT-PCR analysis. These results suggested that the inhibition of melanin exerted by coumaric acid- and caffeic acid-conjugated peptides might provide important information for the development of pigmentation-related skin diseases and cosmetic products.

## Introduction

Melanin is a dark-colored pigment synthesized in melanocytes and transported in vesicles called melanosomes. Melanin is a very important regulator of biological functions; it controls the color of skin, eyes, hair, and other tissues; it also controls skin homeostasis (Slominski et al., 2012). Melanin is carried in melanosomes, which migrate from melanocytes to adjacent keratinocytes. This allows melanin to spread throughout the epidermis, where it protects the skin from environmental exposure, such as harmful ultraviolet (UV) radiation (Epstein, 1983; Menon and Kligman, 2009). Excessive skin melanin production can cause diseases, like skin cancer; thus, melanin abundance is closely related to quality of life. Therefore, in addition to cosmetic considerations, the prevention and treatment of skin pigmentation can improve the quality of life.

In recent years, various methods have become available for preventing and treating skin pigmentation, including surgical procedures (chemical peeling and laser treatment), drugs, and cosmetics (Levy and Emer, 2012; Saxena et al., 2015; Pavlic et al., 2018). Hydroquinone drugs have been mainly used for improving skin pigmentation; antimelanogenic agents including retinol have been used in various combinations, but many ingredients cause side effects when placed on the skin, such as burning, redness, allergy, and cancer (Jow and Hantash, 2014). The cosmetic industry is also developing products that contain various ingredients with skin whitening effects, such as arbutin and kojic acid, to improve pigmentation (Cabanes et al., 1994; Maeda and Fukuda, 1996; Hu et al., 2009). To minimize the adverse effects of placing these products on the skin, the industry has increased the development of cosmetics that include natural products and peptides (Zhang and Falla, 2009; Saewan and Jimtaisong, 2015; Pai et al., 2017; Zaidi et al., 2019).

Coumaric acid and caffeic acid are active ingredients derived from various plants and propolis, which are known to inhibit cellular melanin production in melanocytes by inhibiting signaling pathways related to tyrosinase and the microphthalmia-associated transcription factor (MITF) (Seo et al., 2011; Song et al., 2011; Lee J.Y. et al., 2013; Maruyama et al., 2018). A recent study showed that coumaric acid- and caffeic acid-peptide conjugates could potentially inhibit melanin synthesis (Lee et al., 2012). These conjugated peptides acted as antagonists against the melanocortin receptor 1 (MC1R), a membrane receptor in melanocytes that binds to alpha-melanocyte stimulating hormone (α-MSH) and activates adenylate cyclase (Lee et al., 2012).

In this study, we synthesized coumaric acid- and caffeic acid-peptide conjugates with a mini-PEG or Gly-Gly-Gly (GGG) linker inserted between the acid and the peptide. We determined the effects of these coumaric acid- and caffeic acid-linker-peptide conjugates by measuring cell viability, α-MSH-induced melanin content, tyrosinase activity, and the expression of mRNAs related to genes that control skin melanin synthesis, in human melanoma SK-MEL-2 cells. Our results demonstrated that coumaric acid- and caffeic acid-linker-peptide conjugates had potent catabolic effects on melanin production in human skin melanoma cells. These peptides suppressed the expression of genes related to melanin synthesis, melanin content, and tyrosinase activity. In particular, among nine tested conjugates, the coumaric acid-Gly-Gly-Gly-linker conjugated to the tripeptide, ARP, had the strongest inhibitory effects on melanin synthesis among all synthesized coumaric acid- and caffeic acid-peptide conjugates. Based on these results, we expect that coumaric acid-Gly-Gly-Gly linker-ARP conjugates could represent a new strategy for the development of skin whitening cosmetics and medicinal agents that target melanin synthesis in skin.

## Materials and Methods

### Materials

Coumaric acid- and caffeic acid-peptide conjugates were synthesized by KY Park, a principal researcher of CHA Meditech Co., Ltd (Korea). The human melanoma cell line, SK-MEL-2 (KCLB No. 30068), was purchased from the Korean Cell Line Bank. Fetal bovine serum (FBS), Roswell Park Memorial Institute (RPMI) 1640 cell culture medium, and antibiotics (100 U/ml penicillin and 100 μg/ml streptomycin) were purchased from Corning Inc (USA). The Tyrosinase Inhibitor Screening Kit (Colorimetric) was purchased from BioVision, Inc. (USA). The Alpha-melanin stimulating hormone (α-MSH) was purchased from Sigma Aldrich (USA). Antibodies against phospho-cAMP-response element binding protein-1 (p-CREB-1; sc-81486), microphthalmia-associated transcription factor (MITF; sc-515925), tyrosinase (TYR; sc-20035), horseradish peroxidase (HRP)-conjugated secondary antibodies, and HRP-conjugated actin were purchased from Santa Cruz Biotechnology, Inc. (USA).

### Cell Culture and Cell Viability

Human melanoma SK-MEL-2 cells were maintained in RPMI 1640 medium containing 10% FBS and 1% antibiotics in a humidified atmosphere of 5% CO_2_ at 37°C. Human skin normal fibroblast CCD-986sk cells were maintained in Iscove's Modified Dulbecco's Medium (IMEM) containing 10% FBS and 1% antibiotics in a humidified atmosphere of 5% CO_2_ at 37°C. To determine cell viability, SK-MEL-2 cells or CCD-986sk cells (5 × 10^3^ cells/well) were seeded in a 96-well plate for 24 h, then treated with peptides for 24 h in culture media. Cell viability was assessed with the Cell Counting Kit-8 (Dojindo Molecular Technologies, Japan), according to manufacturer instructions. The absorbance was measured with a Multiscan™ FC microplate photometer (Thermo Fisher Scientific, USA).

### Melanin Content

SK-MEL-2 cells (5 × 10^5^ cells/ml) were seeded in 60 mm^2^ dishes and incubated for 24 h. Cells were treated with α-MSH (200 nM) and either arbutin or peptides in culture medium containing 10% FBS for 72 h. After incubation, cells were harvested, washed with PBS, resuspended in 1 N NaOH containing 10% DMSO, and heated at 80°C for 1 h. The absorbance of extracted melanin in cell lysates was measured with a Multiscan™ FC microplate photometer (Thermo Fisher Scientific, USA) at 405 nm.

### Tyrosinase Activity Test

To measure tyrosinase activity in peptide-treated cells, SK-MEL-2 cells (5 × 10^4^ cells/well) were seeded in 6-well plates and incubated for 24 h. Cells were treated with α-MSH (200 nM) and arbutin or peptides for 72 h. Arbutin, a known tyrosinase inhibitor, was used as the positive control. Tyrosinase activity in peptide-treated cells or tyrosinase activity *in vitro* was measured with the Tyrosinase Inhibitor Screening Kit (colorimetric) (BioVision, Inc., USA); the absorbance was measured with a Multiscan™ FC microplate photometer (Thermo Fisher Scientific, USA).

### Quantitative Real-Time PCR Analysis

SK-MEL-2 cells were treated with α-MSH (200 nM) and either arbutin or peptides in culture medium containing 10% FBS. Total RNA was isolated with the AccuPrep^®^ RNA Extraction Kit (Bioneer Corp., Korea). Next, total RNA (1 μg) was reverse transcribed to obtain cDNA with the RocketScript™ Reverse Transcriptase Kit (Bioneer Corp., Korea) and oligo (dT) primers (Bioneer Corp., Korea). Quantitative real-time PCR (qRT-PCR) was performed with the ExcelTaq 2X Q-PCR Master Mix (SMOBiO, Taiwan) and the CFX96™ Real-Time System (Bio-Rad, USA). Thermocycling conditions were: 95°C for 3 min, followed by 40 cycles at 95°C for 15 s, 60°C for 30 s, and 72°C for 30 s. The primer sequences used in this study are shown in **Supplementary Table S1**. All reactions were run in triplicate, and relative expression levels were determined with the 2^−ΔΔC^_T_ method (Livak and Schmittgen, 2001). GAPDH was used as the internal standard.

### Western Blot

SK-MEL-2 cell lysates were prepared using NE-PER nuclear and cytoplasmic extraction reagent. After protein quantification, lysates (40 μg) were resolved by SDS-PAGE and transferred to nitrocellulose membranes. The membranes were labeled with primary antibodies for specific protein detection, and then incubated with HRP-conjugated secondary antibodies. The protein expression was visualized using SuperSignal™ West Femto Maximum Sensitivity Substrate (Thermo Fisher Scientific, USA). The band intensities were measured using X-ray films and development solution (Fujifilm, Tokyo, Japan).

### Statistical Analysis

Data are presented as the mean ± SD of at least three independent experiments. Significant differences between groups were evaluated with the one-way ANOVA and *post-hoc* Tukey HSD test. Significant differences in relative gene expression levels were evaluated with the Student's *t*-test, based on GAPDH-normalized, 2^−ΔΔC^_T_ values (Livak and Schmittgen, 2001). P-values <0.05 were considered significant.

## Results

### Synthesis of Coumaric Acid- and Caffeic Acid-Conjugated Peptides

**Figure 1:**
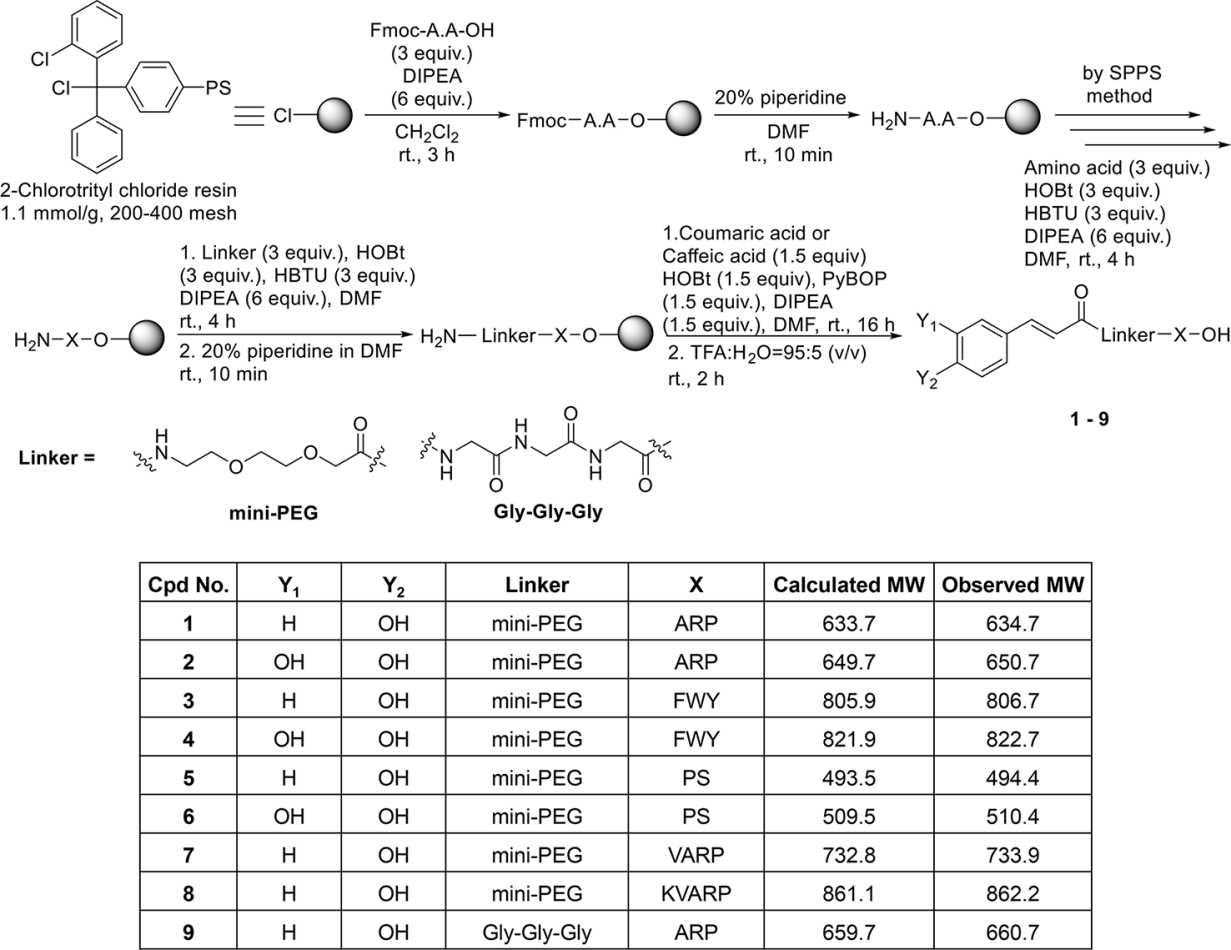
Solid phase synthesis of coumaric acid- and caffeic acid-peptide conjugates.

Coumaric acid- and caffeic acid-conjugated peptides (1–9) were synthesized with the solid phase peptide synthesis method (**Figure 1**). The entire coupling reaction for peptide synthesis was carried out with HOBt/HBTU/DIPEA reagents. Fmoc-miniPEG was used as a linker, inserted between the acid and the peptide. The small molecule compounds (coumaric acid and caffeic acid) were synthesized as described previously (Koskinen et al., 1995).

The coupling reaction between the peptides bound to the 2-Cl-trityl resin and the small molecule compounds was carried out with PyBOP/HOBt/DIPEA. Finally, the resin was treated with a cleavage cocktail (TFA/H_2_O = 95:5 v/v) for 2 h. **Supplementary Figures S1–18** show the characterization of the coumaric acid- and caffeic acid-conjugated peptides (1-9). Purity (%) of all compounds is indicated as “Area %” in the HPLC spectrum peak tables of **Supplementary Figures S1–18**.

### Cytotoxicity of Coumaric Acid- and Caffeic Acid-Conjugated Peptides

**Figure 2:**
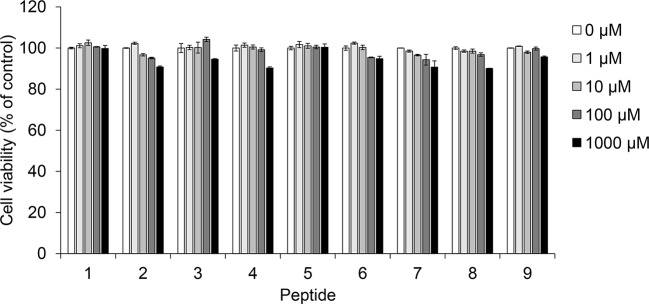
The effect of coumaric acid- and caffeic acid-peptide conjugates on the viability of SK-MEL-2 cells. SK-MEL-2 cells were treated with coumaric acid- or caffeic acid-conjugated peptides (**1**–**9**) for 72 h. Cell viability is expressed as the percent of control (control: 0 μM). Experiments were performed in triplicate. Statistical significance is represented in **Supplementary Table S2**.

Prior to confirming melanin synthesis inhibition, we checked the cytotoxicity of coumaric acid- and caffeic acid-conjugated peptides in SK-MEL-2 cells (**Figure 2**). We observed no significant cytotoxicity with coumaric acid- or caffeic acid-conjugated peptides (1-9), even at the 1 mM concentration.

### Melanogenesis Inhibition by Coumaric Acid- and Caffeic Acid-Conjugated Peptides

**Figure 3:**
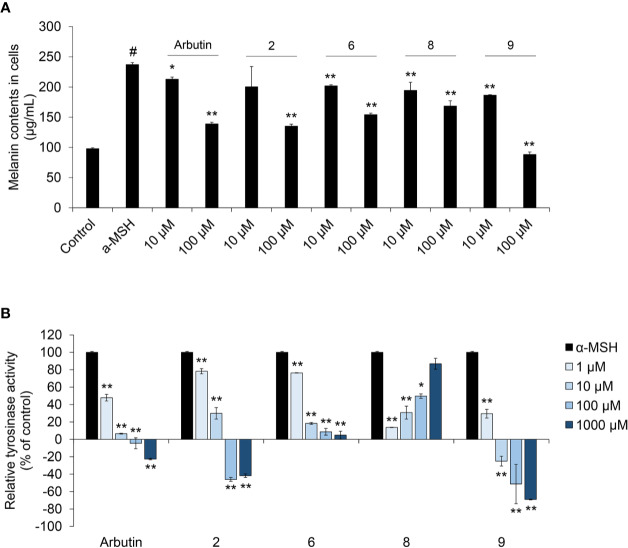
The effect of coumaric acid- and caffeic acid-peptide conjugates on α-MSH –induced melanin synthesis and tyrosinase activity in SK-MEL-2 cells. SK-MEL-2 cells were treated with α-MSH (200 nM) alone or with the indicated concentrations of peptides for 72 h. **(A)** Melanin content in cells (μg/ml). **(B)** Relative tyrosinase inhibition is expressed as a percentage of control (control: α-MSH only). Data represent the mean ± SD of experiments performed in triplicate. ^#^*p* < 0.01 *versus* control, ^*^p < 0.05, ^**^p < 0.01 *versus* treatment with α-MSH alone.

To assess the anti-melanogenic effects of coumaric acid- and caffeic acid-conjugated peptides, we measured the α-MSH-induced melanin content in SK-MEL-2 cells (**Figure 3A**). Arbutin was used as a positive control, because it was known to inhibit melanogenesis. Among all the synthesized coumaric acid- and caffeic acid-conjugated peptides, four (2, 6, 8, and 9) showed enhanced inhibitory effects compared to the effect of arbutin. These peptides did not show significant cytotoxicity in human normal skin fibroblasts (**Supplementary Figure S19**). The α-MSH treatment increased melanin content by about 150%, compared to the control (untreated) group. However, at 10 μM and 100 μM, arbutin and the conjugated peptides inhibited the α-MSH-induced melanin synthesis. Moreover, both coumaric acid alone and a mixture of coumaric acid and the ARP peptide showed inhibitory effects on melanin synthesis, but they were not as effective as compound 9, which included a GGG linker (**Supplementary Figure S20**).

In melanocytes, melanin synthesis is catalyzed by tyrosinase in melanosomes (Boo, 2019). Therefore, controlling tyrosinase activity is useful strategy for inhibiting melanin synthesis in skin. Accordingly, we measured the tyrosinase activity in cells treated with α-MSH and peptides. We found that arbutin and four of our synthesized peptides inhibited tyrosinase activity (**Figure 3B** and **Supplementary Figure S21**). In particular, dose-dependent inhibition was observed with compounds 2, 6, and 9; moreover, compound **9** exerted stronger inhibition than arbutin. These results indicated that tyrosinase inhibition by coumaric acid- and caffeic acid-conjugated peptides could influence melanin synthesis in SK-MEL-2 cells.

### Melanin-Related Gene Expression Regulated by Coumaric Acid- and Caffeic Acid-Conjugated Peptides

During melanogenesis, α-MSH is a physiological ligand that binds MC1R, which activates cyclic AMP (cAMP) production (Boo, 2019). Cyclic AMP activates cAMP-dependent protein kinase A (PKA) and promotes the expression of *MITF*, tyrosinase (*TYR*), tyrosinase-related protein-1 (*TRP-1*), and tyrosinase-related protein-2 (*TRP-2*) in the melanocyte nucleus (Aoki and Moro, 2012; Slominski et al., 2004; Boo, 2019).

**Figure 4:**
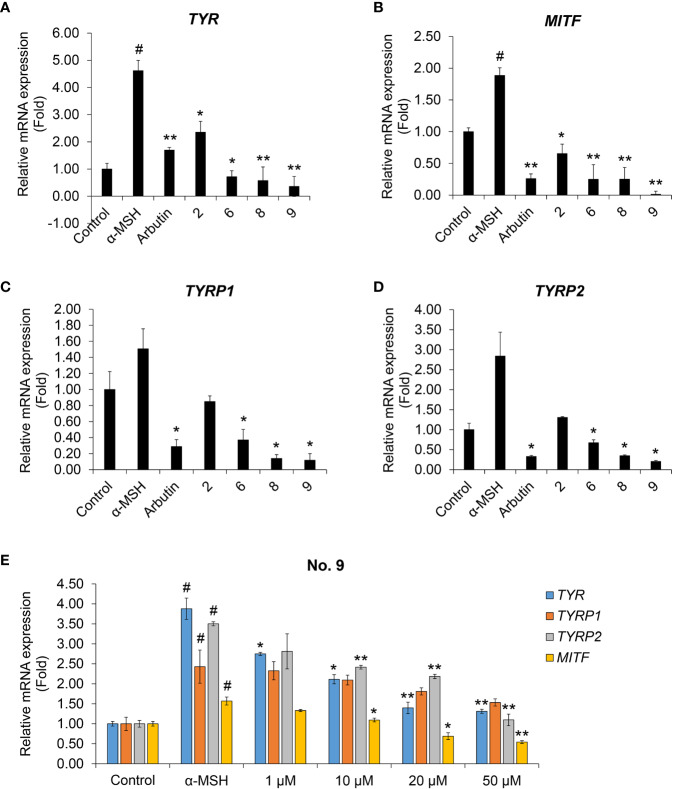
The effects of coumaric acid- and caffeic acid-peptide conjugates on the expression of melanogenesis-related genes. **(A–D)** Relative gene expression, expressed as the fold change in mRNA levels compared to control (untreated) in SK-MEL-2 cells. Cells were treated with α-MSH (200 nM), without or with 100 μM of coumaric acid- or caffeic acid-conjugated peptides (2, 6, 8, 9) or arbutin for 48 h. **(E)** Relative gene expression, expressed as the fold change in mRNA levels in SK-MEL-2 cells, compared to control (untreated) cells. Cells were treated with α-MSH (200 nM) without or with different concentrations of compound 9 (1–50 μM) for 48 h. GAPDH served as the internal control. Data represent the mean ± SD of experiments performed in triplicate. ^#^*p* < 0.01 *versus* control, ^*^p < 0.05, ^**^p < 0.01 *versus* treatment with α-MSH only.

We performed real-time PCR to investigate the effects of coumaric acid- and caffeic acid-conjugated peptides on the levels of mRNAs transcribed from melanogenesis-related genes (**Figure 4**). We found that treating with α-MSH increased the expression of *TYR*, *TYRP1*, *TYRP2*, and *MITF* genes. However, arbutin and four conjugated peptides suppressed the expression of these genes. In particular, compound 9 showed stronger inhibition than arbutin and the other peptides.

We then tested increasing concentrations of compound **9** to evaluate dose-response effects on the levels of *TYR*, *TYRP1*, *TYRP2*, and *MITF* mRNAs (**Figure 4E**). We found that compound 9 dose-dependently suppressed the mRNA expression of melanin synthesis-related genes. In addition, compound 9 inhibited the protein expression of MITF and TYR and phosphorylation of the upstream mediator CREB in melanin production process (**Supplementary Figure S22**). This result indicated that the coumaric acid (or caffeic acid)-GGG linker, conjugated with ARP peptide, had a strong inhibitory effect on transcriptional activity and protein expression related to pigmentation in skin melanocytes, and that these effects exceeded the effects of arbutin treatment alone.

## Discussion

Melanogenesis is a complex, multi-step process that involves a variety of physiological functions in human skin. For example, exposure to UV radiation stimulates keratinocytes to secrete α-MSH in melanosomes, which are then delivered to the epidermis (Chakraborty et al., 1995). Released α-MSH binds to the MC1R to activate cAMP production, which in turn promotes downstream signaling (Boo, 2019). During this process, MITF is a key transcription factor that regulates the transcription of melanogenesis-inducing enzymes, such as TYR, TRP-1, and TRP-2 (Yasumoto et al., 1997; Bertolotto et al., 1998). Accordingly, regulating α-MSH binding to MC1R and suppressing MITF transcriptional activity could be effective strategies for controlling skin pigmentation.

Many previous studies have shown the potential of coumaric acid and caffeic acid as anti-melanogenic agents. The chemical structures of coumaric acid and caffeic acid are very similar to that of L-tyrosine. Therefore, these natural substrates can be degraded by tyrosinase, and thus, they can act as competitive inhibitors in melanin production (Lee et al., 2012; Lee J.Y. et al., 2013; Lee H.S. et al., 2013; Maruyama et al., 2018). Consequently, coumaric acid and caffeic acid might be natural antagonists to α-MSH-induced melanogenesis in melanocytes. Indeed, recent reports have shown that coumaric acid- and caffeic acid-peptide conjugates could serve as skin-whitening agents (Lee et al., 2012; Lee J.Y. et al., 2013). They also showed that both coumaric acid and caffeic acid conjugates inhibited melanin production more effectively than arbutin and kojic acid. In particular, almost all compounds that had the –AR– peptide sequence showed anti-melanogenic effects superior to the effects achieved with coumaric acid or caffeic acid (Lee J.Y. et al., 2013; Lee H.S. et al., 2013).

Based on those findings, we also synthesized coumaric acid- and caffeic acid-peptide conjugates. However, unlike the previous studies, we used linkers to conjugate coumaric acid or caffeic acid with peptides. We tested two types of linkers: mini-polyethylene glycol (PEG) and Gly-Gly-Gly (GGG). These linkers were previously used to enhance the uptake of potent imaging probes in melanoma-bearing mice (Guo and Miao, 2014). In the present study, we bonded short peptides to coumaric acid- or caffeic acid-linker conjugates. Among these conjugated peptides, coumaric acid-GGG-ARP (compound 9) showed the strongest inhibition of melanin synthesis and melanin-related gene expression in human melanoma cells. The use of a linker, rather than directly linking coumaric acid to a peptide, improved the physical properties of the compound, which increased its efficacy in inhibiting melanin production. In addition, the coumaric acid was dissolved only in DMSO, but the use of a linker has the advantage of increasing the solubility of coumaric acid-GGG-ARP in water, thereby increasing the efficacy in cells.

Many studies have shown that short peptides or peptidomimetics could act as potent tyrosinase inhibitors (Pillaiyar et al., 2018). For example, an octapeptide P3 (Arg-Ala-Asp-Ser-Arg-Ala-Asp-Cys) and the decapeptide P4 (Tyr-Arg-Ser-Arg-Lys-Tyr-Ser-Ser-Trp-Tyr) could inhibit human or mouse tyrosinase activities in melanocytes, without cytotoxicity (Reddy et al., 2012). In addition, many types of peptides and conjugates have emerged as effective cosmetic agents that could improve skin pigmentation. Our results also provided useful information for the development of skin whitening cosmetics or related products. Recently, we applied for a patent, based on the results of this study.

In conclusion, we confirmed that coumaric acid-linker-peptide conjugates had inhibitory effects on melanin synthesis, tyrosinase activity, and melanin-related gene expression. In future, the detailed mechanisms of skin pigmentation signaling pathways should be studied. In the near future, we plan to incorporate the coumaric acid-GGG-ARP conjugate into skin pigmentation prevention products, such as cosmetics and medical supplies, and evaluate the effects of inhibiting melanin synthesis.

## Data Availability Statement

All datasets generated for this study are included in the article/**Supplementary Material**.

## Ethics Statement

This study was conducted by culturing an existing cell line. We did not use any animals or human specimens.

## Author Contributions

K-YP and JK collected and analyzed the background research. K-YP synthesized the compounds and JK created the figures. JK wrote the manuscript.

## Conflict of Interest

K-YP was employed by the company CHA Meditech Co., Ltd.

The remaining author declares that the research was conducted in the absence of any commercial or financial relationships that could be construed as a potential conflict of interest.
