# Supplementary material for: Synthesis and Biological Evaluation of the Anti-Melanogenesis Effect of Coumaric and Caffeic Acid-Conjugated Peptides in Human Melanocytes
Source: Front Pharmacol. 2020 Jun 17;11:922. doi: 10.3389/fphar.2020.00922 (PMC7311773; doi:10.3389/fphar.2020.00922)
Supplement: Supplementary file 1 [file DataSheet_1.docx]

**Experimental Section**

**Characterization of coumaric and caffeic acid-conjugated peptides**

**Mass spectrometry**

Coumaric and caffeic acid-conjugated peptide masses were determined on Waters ACQUITY UPLC H-Class/SQD2

**HPLC spectrometry**

HPLC analysis of coumaric acid and caffeic acid-conjugated peptides was conducted in H_2_O solution (1 mg/ml) using a Shimadzu HPLC 2030 model. Analysis conditions are as follows

| - Instrument | : Shimadzu HPLC 2030 system |
| --- | --- |
| - Flow Rate | : 1 ml/min |
| - Gradient | : 0%-03% B in 03 min; 03%-60% B in 33 min;60% B in 35 min |
| - Buffer | : A buffer 0.1% TFA in H_2_O; B buffer 0.1% TFA in CH_3_CN |
| - Column | : Vydac 218TP C18, 5 um, 4.6 X 250 mm |
| - Yields | : 70~80% |


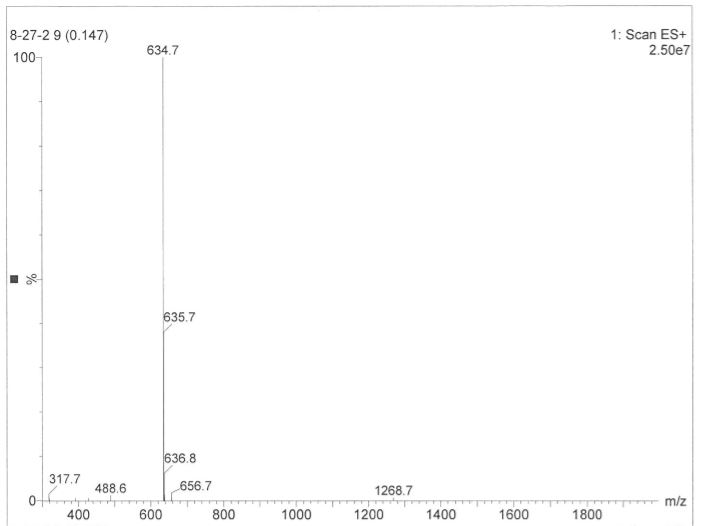


**Figure S1.** LC-Mass spectrum of **1** compound.


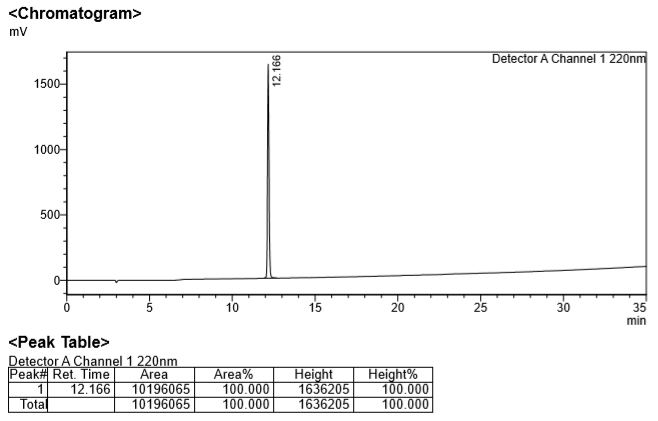


**Figure S2.** HPLC spectrum of **1** compound.


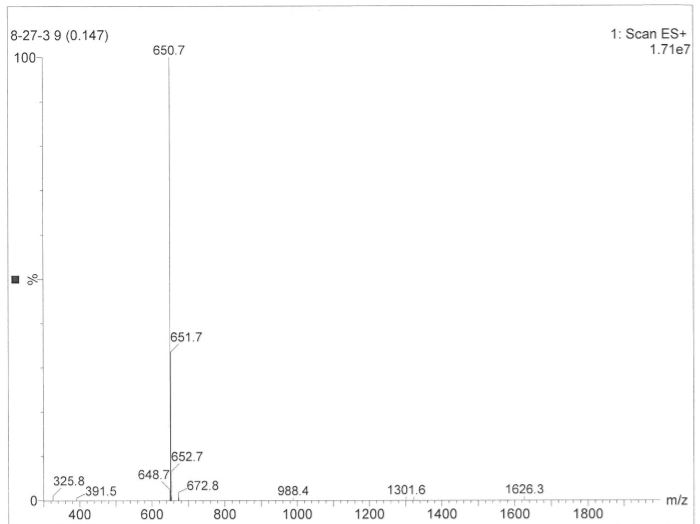


**Figure S3.** LC-Mass spectrum of **2** compound.


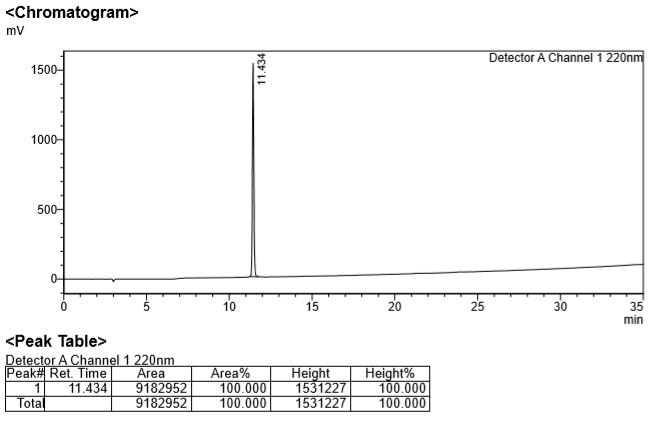


**Figure S4.** HPLC spectrum of **2** compound.


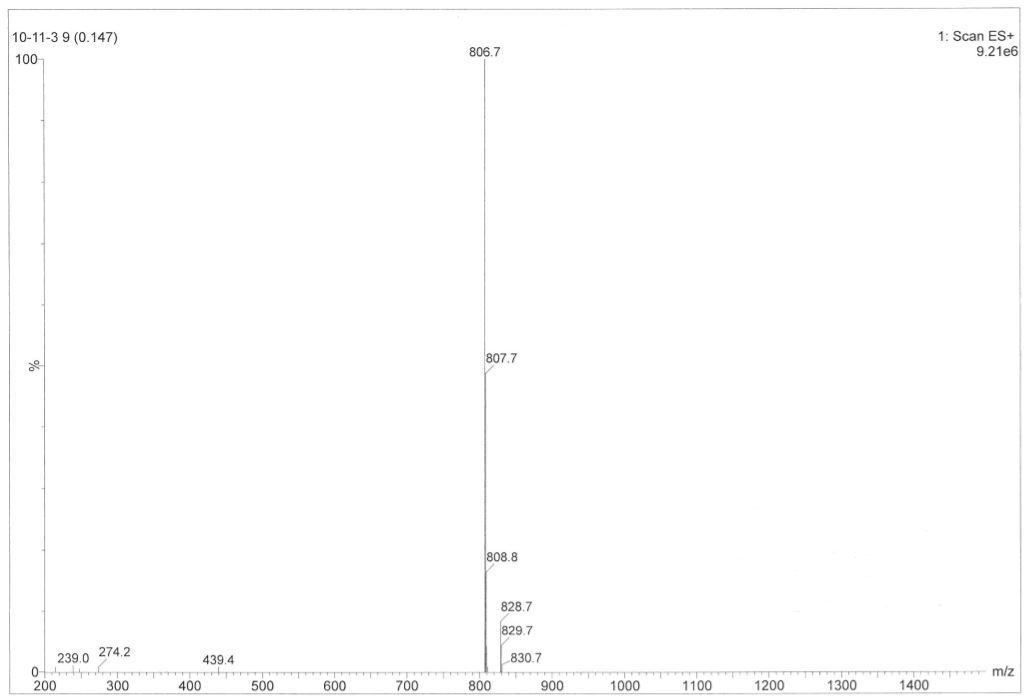


**Figure S5.** LC-Mass spectrum of **3** compound.


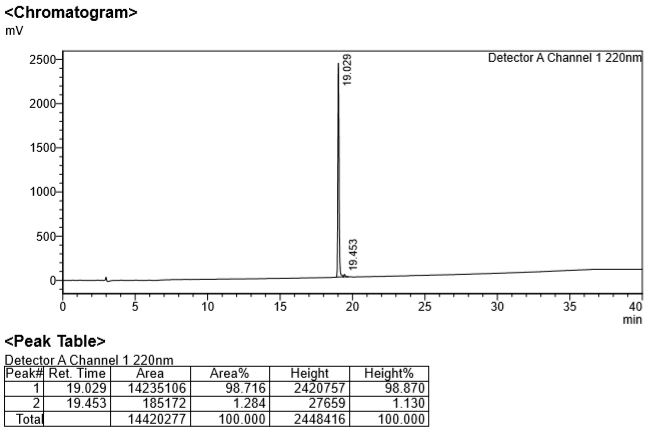


**Figure S6.** HPLC spectrum of **3** compound.


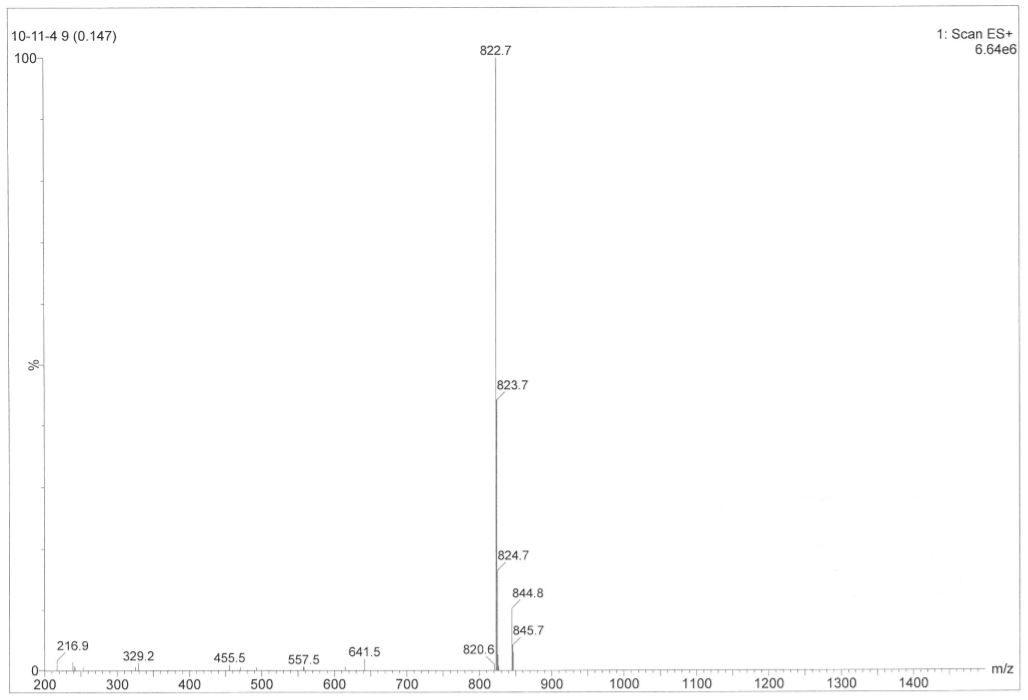


**Figure S7.** LC-Mass spectrum of **4** compound.


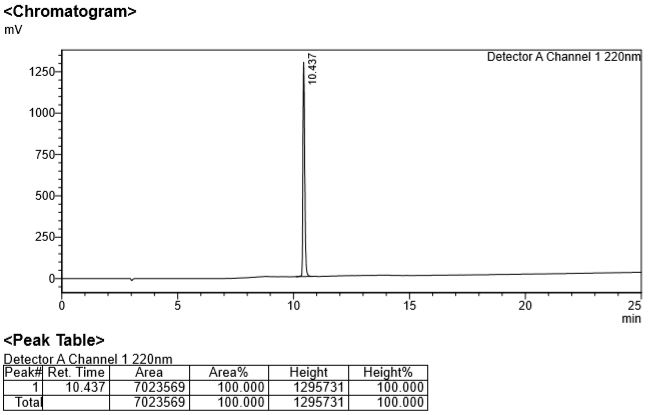


**Figure S8.** HPLC spectrum of **4** compound.


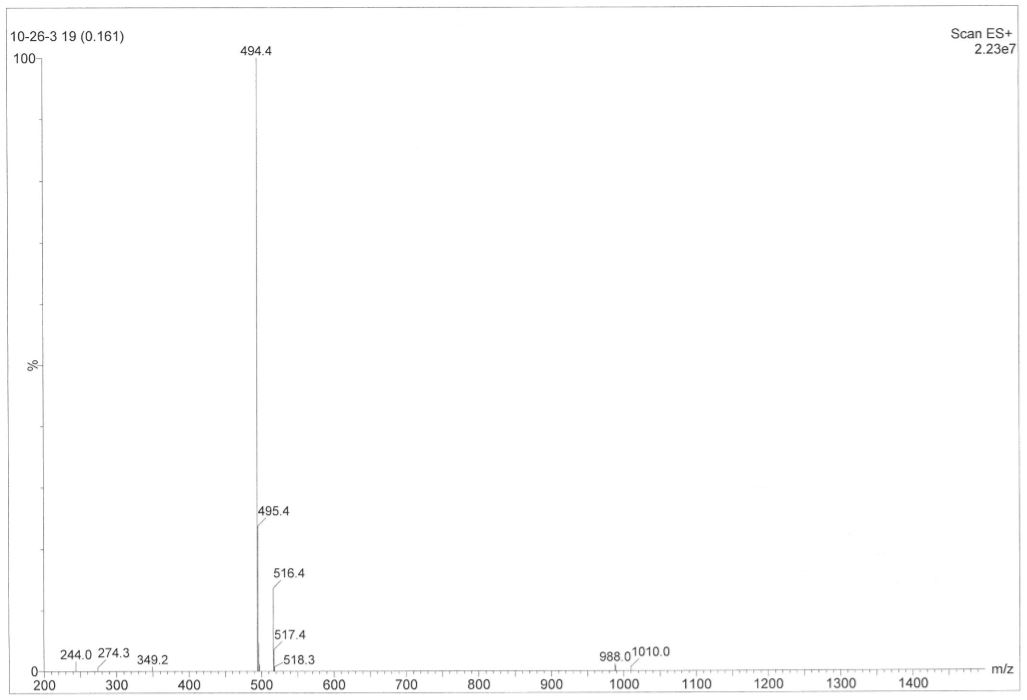


**Figure S9.** LC-Mass spectrum of **5** compound.


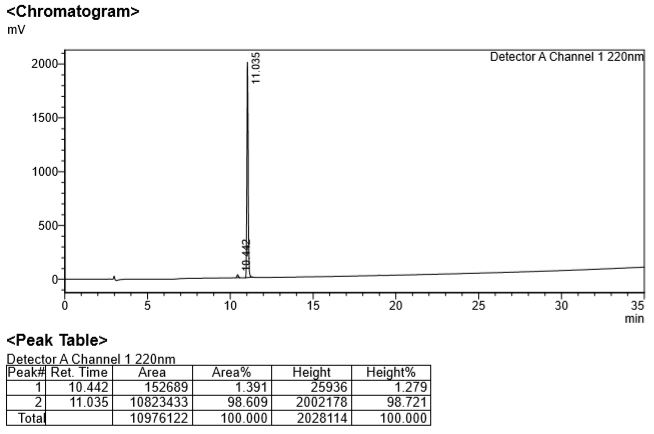


**Figure S10.** HPLC spectrum of **5** compound.


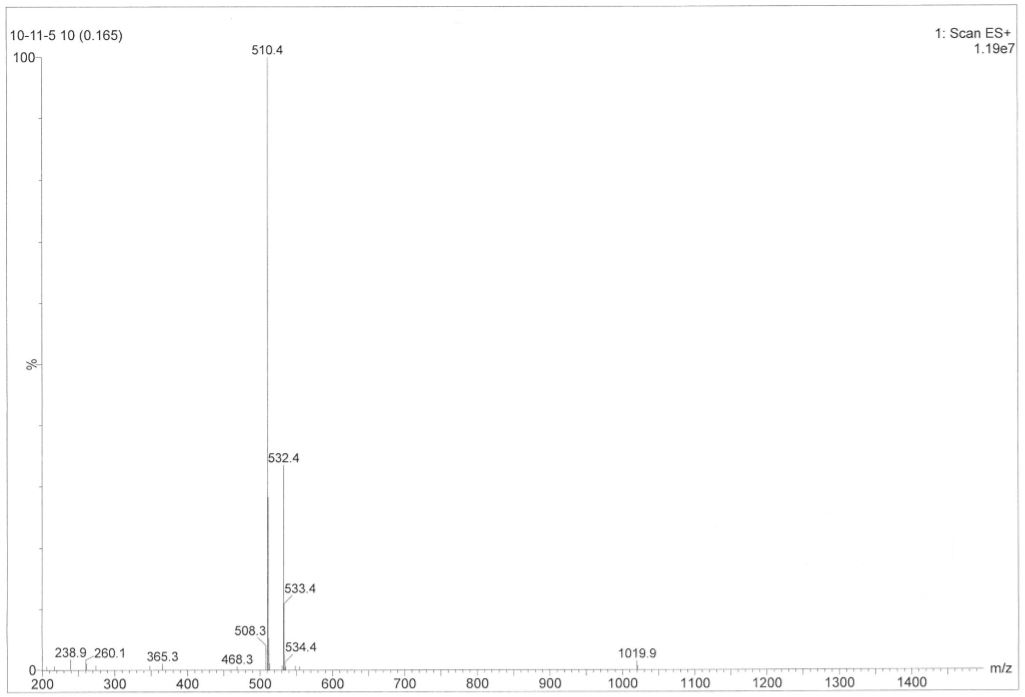


**Figure S11.** LC-Mass spectrum of **6** compound.


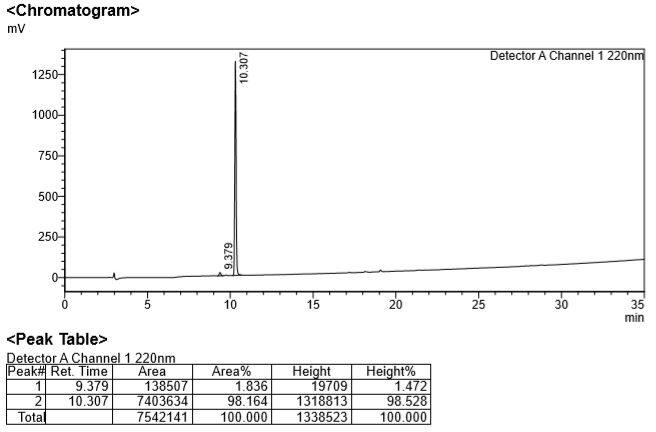


**Figure S12.** HPLC spectrum of **6** compound.


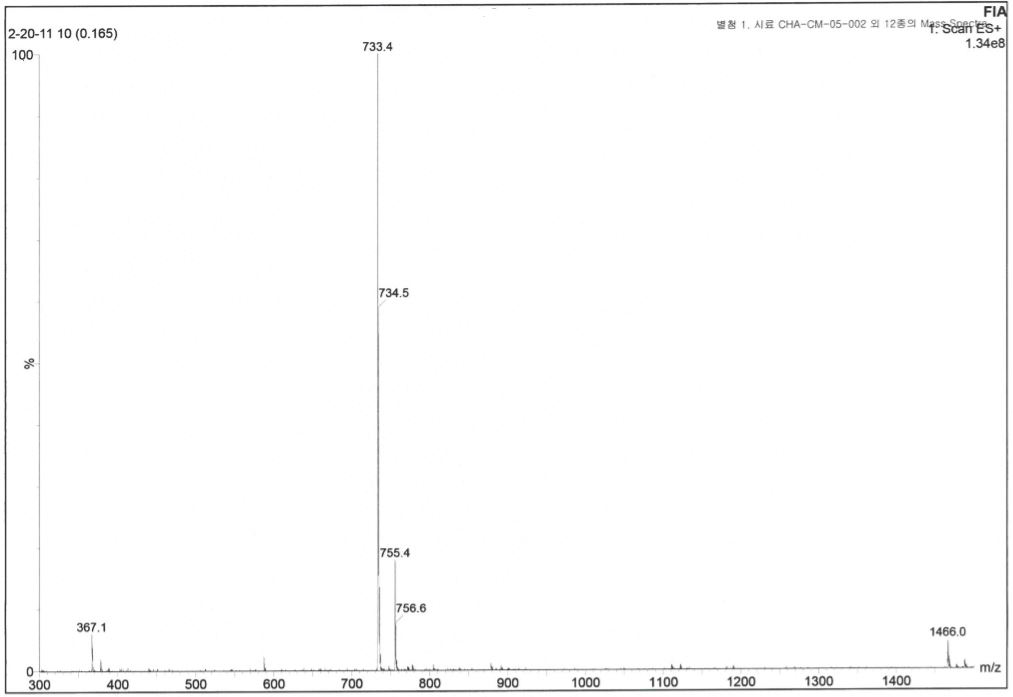


**Figure S13.** LC-Mass spectrum of **7** compound.


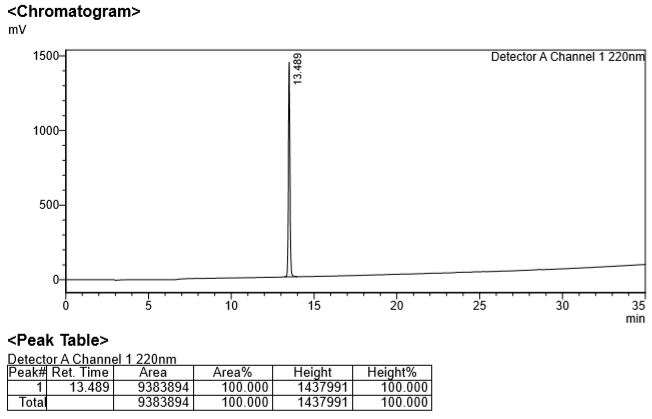


**Figure S14.** HPLC spectrum of **7** compound.


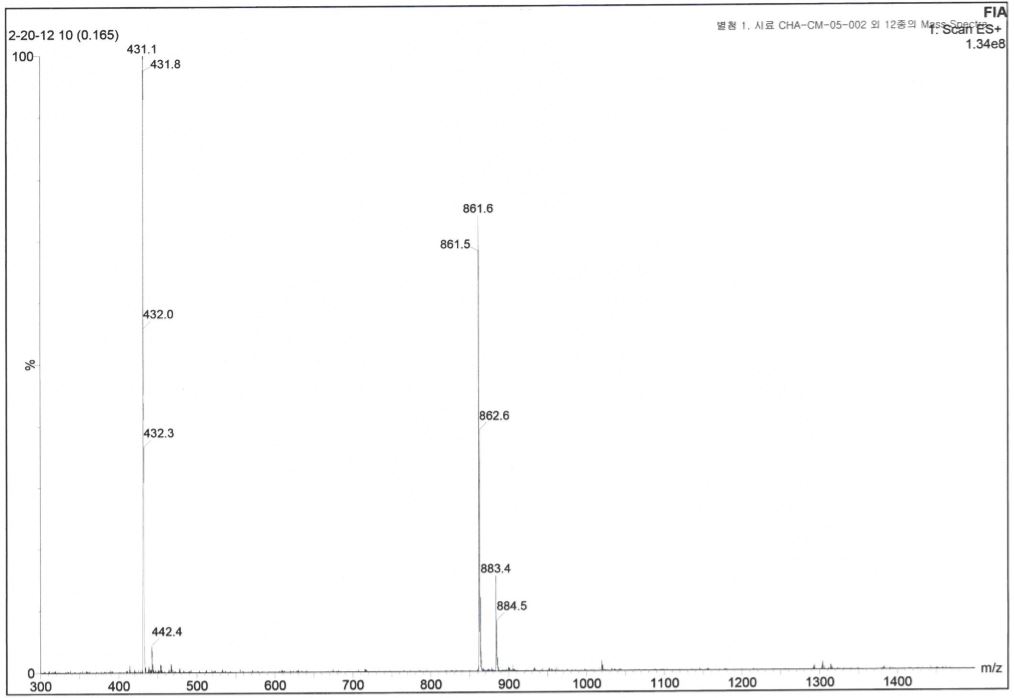


**Figure S15.** LC-Mass spectrum of **8** compound.


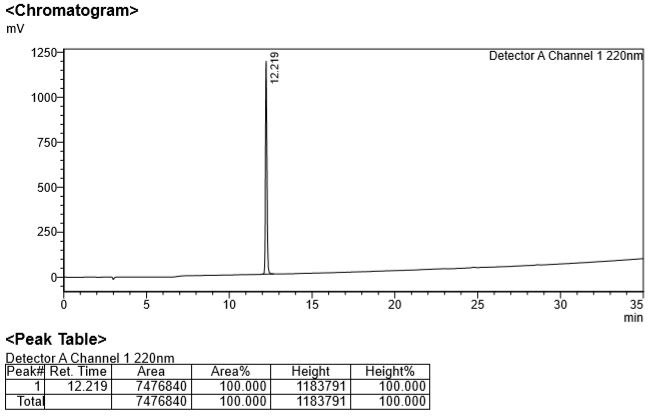


**Figure S16.** HPLC spectrum of **8** compound.


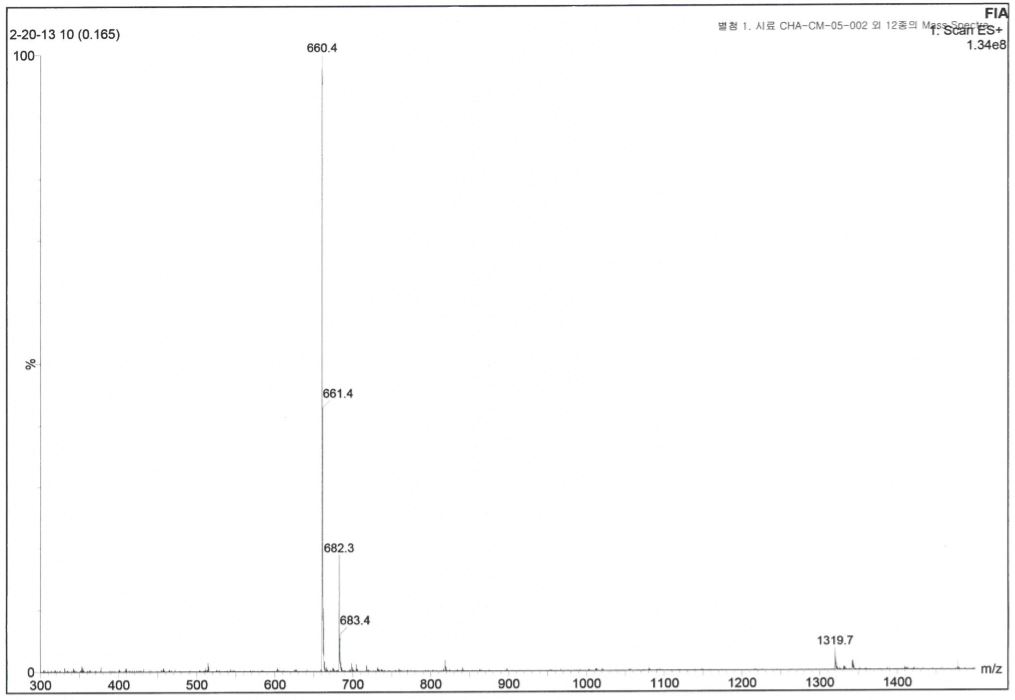


**Figure S17.** LC-Mass spectrum of **9** compound.


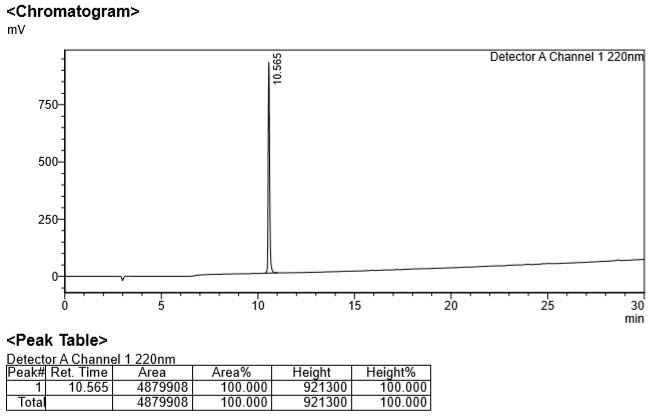


**Figure S18.** HPLC spectrum of **9** compound.

**
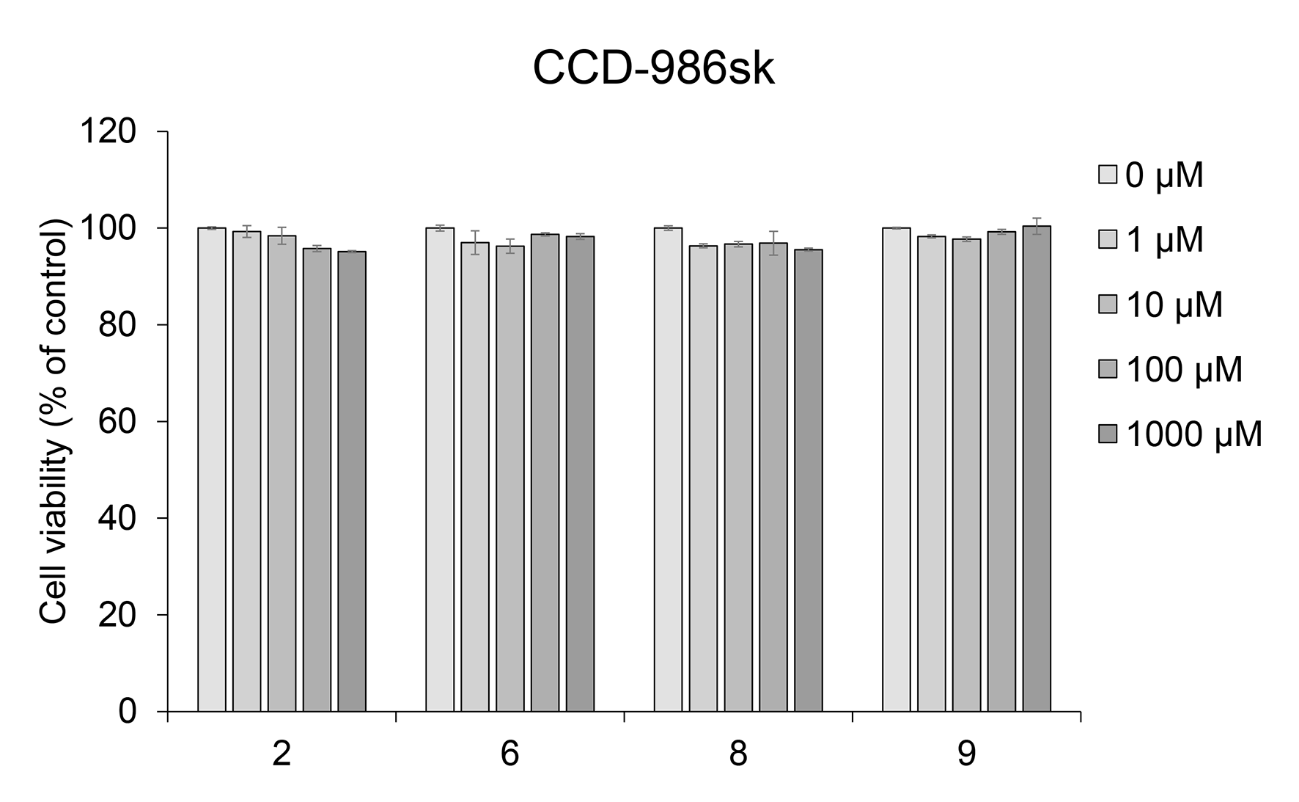
**

**Figure S19.** The effect of coumaric acid- and caffeic acid-peptide conjugates on the viability of human skin fibroblast CCD-986sk cells. CCD-986sk cells (passage 12) were treated with coumaric acid- or caffeic acid-conjugated peptides (Cpd No. **2**, **6**, **8** and **9**) for 72 h. Cell viability is expressed as the percent of control (control: 0 μM). Experiments were performed in triplicate.


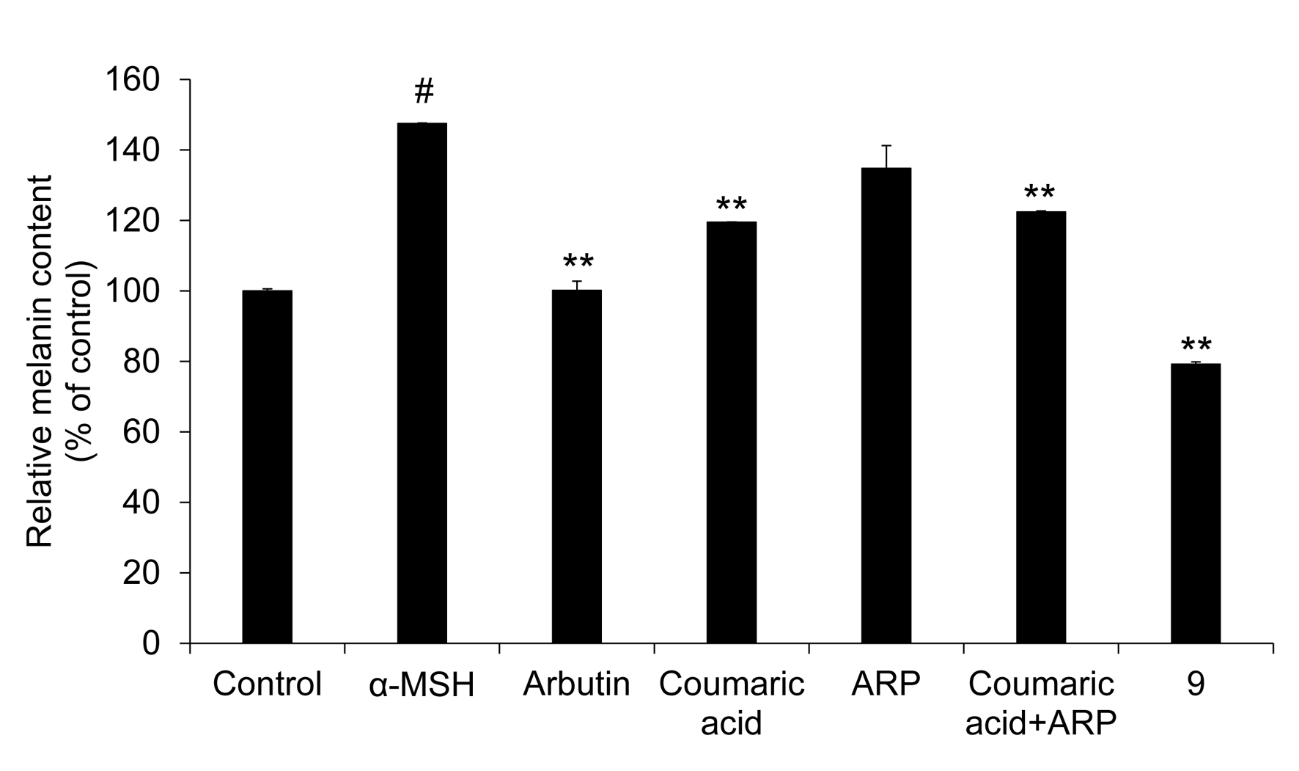


**Figure S20.** The effect of coumaric acid and tripeptide ARP on α-MSH –induced melanin synthesis in SK-MEL-2 cells. SK-MEL-2 cells were seeded on 6-well plates for 24 h. After incubation, cells were treated with α-MSH (200 nM) and arbutin (100 μM) or coumaric acid (100 μM) or ARP (100 μM) or combination of coumaric acid and ARP (100 μM) or cpd **9** (100 μM) for 72 h. After incubation, cells were washed twice with PBS, resuspended in 1 N NaOH containing 10% DMSO, and heated at 80 °C for 1 h. The absorbance of extracted melanin and was measured by an ELISA microplate reader at 405 nm. Detailed experimental procedures are described in the Materials and Methods. Data represents mean ± SD. Experiments were performed in triplicate. ^#^ *p* < 0.01 versus control, ^*^ *p* < 0.05, ^**^ *p* < 0.01 versus treatment with α-MSH only.


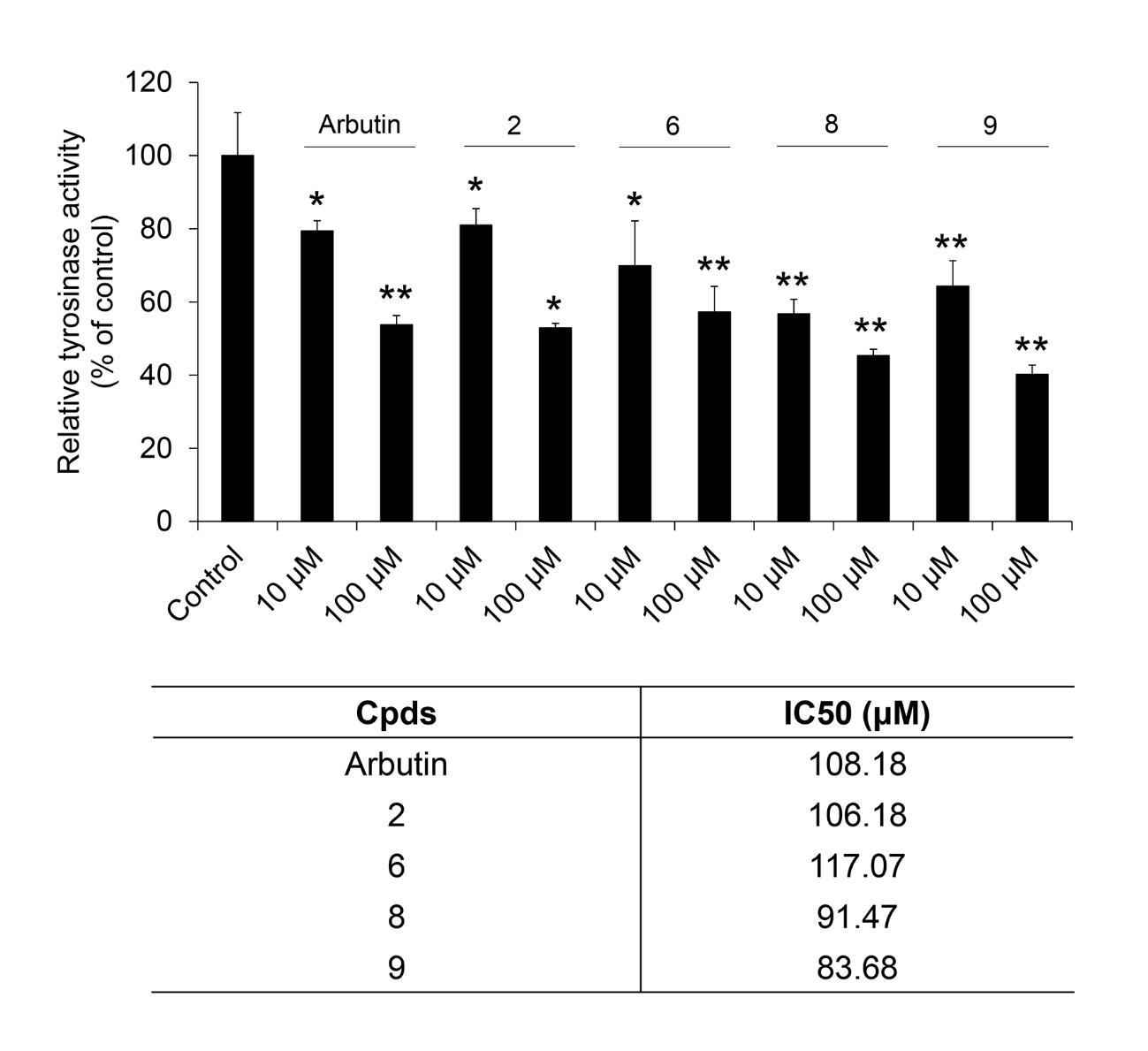


**Figure S21.** The effect of coumaric acid- and caffeic acid-peptide conjugates on *in vitro* tyrosinase activity. Relative tyrosinase inhibition is expressed as a percentage of control (control: tyrosinase substrate and tyrosinase treated group). Detailed experimental procedures were described in Materials and Methods section. Data represent the mean ± SD of experiments performed in triplicate. ^*^ *p* < 0.05, ^**^ *p* < 0.01.

**Figure S22.** The effects of compound No.9 on the expression of melanogenesis-related proteins. SK-MEL-2 cells were treated with α-MSH (200 nM) and different concentrations of compound **9** (1 – 50 μM) for 30 min (p-CREB), 6 h (MITF), and 72 h (TYR), respectively. The protein expression was measured by western blot. Detailed experimental procedures were described in Materials and Methods section. Actin was used as a loading control.

| **Target gene** | **Forward (5’ – 3’)** | **Reverse (5’ – 3’)** |
| --- | --- | --- |
| *TYR* | TGCCCCAAGAAGGACAAAT | GTGCTGACCTCCCATGTACT |
| *TYRP1* | ATGGCAGAGATGATCGGGAG | GAGCTTCAACTCCAACCCTT |
| *TYRP2* | AGTACGACAGAGACAAGGAAAGT | ACGGTCATCCTGGTTTCGTA |
| *MITF* | TGAGCATGGAAGAGACGGAG | TCCAAGGAAGTCACAGGCAT |
| *GAPDH* | GAGTCAACGGATTTGGTCGT | GATCTCGCTCCTGGAAGATG |

**Table S1.** Sequences of primers used in this study.

**Table S2.** Effect of coumaric acid or caffeic acid-peptide conjugates on the cell viability of SK-MEL-2 cells

| Cpd No. | Conc. (μM) | Cell viability  (% of control) | P value |
| --- | --- | --- | --- |
| **1** | 1 | 101.12 | 0.1075 |
|  | 10 | 99.62 | 0.6632 |
|  | 100 | 98.34 | 0.0738 |
|  | 1000 | 98.58 | 0.1357 |
| **1** | 1 | 102.53 | 0.1509 |
|  | 10 | 101.57 | 0.1640 |
|  | 100 | 95.06 | 0.0053 |
|  | 1000 | 91.33 | 0.0006 |
| **3** | 1 | 101.12 | 0.1455 |
|  | 10 | 102.57 | 0.0329 |
|  | 100 | 100.62 | 0.0715 |
|  | 1000 | 99.78 | 0.8134 |
| **4** | 1 | 102.29 | 0.0023 |
|  | 10 | 96.73 | 0.0008 |
|  | 100 | 95.17 | 0.0000 |
|  | 1000 | 90.81 | 0.0000 |
| **5** | 1 | 99.96 | 0.9749 |
|  | 10 | 99.92 | 0.9632 |
|  | 100 | 101.22 | 0.5405 |
|  | 1000 | 93.65 | 0.0067 |
| **6** | 1 | 101.44 | 0.5185 |
|  | 10 | 99.40 | 0.6710 |
|  | 100 | 101.25 | 0.4948 |
|  | 1000 | 90.83 | 0.0021 |
| **7** | 1 | 100.29 | 0.8489 |
|  | 10 | 100.22 | 0.9149 |
|  | 100 | 104.22 | 0.0404 |
|  | 1000 | 94.58 | 0.0133 |
| **8** | 1 | 101.42 | 0.2385 |
|  | 10 | 100.51 | 0.6395 |
|  | 100 | 99.19 | 0.4640 |
|  | 1000 | 90.33 | 0.0004 |
| **9** | 1 | 101.95 | 0.4219 |
|  | 10 | 99.64 | 0.8567 |
|  | 100 | 98.32 | 0.4632 |
|  | 1000 | 96.64 | 0.1087 |

**Table S3**. IC50 of coumaric acid or caffeic acid-peptide conjugates on α-MSH-induced melanin synthesis and tyrosinase activity inhibition in SK-MEL-2 cells.

| **Test** | **Cpds** | **IC50 (μM)** |
| --- | --- | --- |
| **Melanin synthesis** | Arbutin | 100.28 |
|  | 2 | 97.62 |
|  | 6 | 112.71 |
|  | 8 | 137.93 |
|  | 9 | 73.35 |
| **Tyrosinase activity inhibition** | Arbutin | 407.50 |
|  | 2 | 352.86 |
|  | 6 | 525.21 |
|  | 8 | - |
|  | 9 | 295.68 |

**Table S4**. IC50 of coumaric acid-GGG-ARP (compound No. 9) on α-MSH-induced mRNA expression of melanin synthesis related genes in SK-MEL-2 cells.

| **Gene** | **IC50 (μM)** |
| --- | --- |
| ***TYR*** | 37.82 |
| ***TYRP1*** | 67.75 |
| ***TYRP2*** | 36.41 |
| ***MITF*** | 38.24 |
